# High-resolution characterization of the forbidden Si 200 and Si 222 reflections^1^

**DOI:** 10.1107/S1600576715004732

**Published:** 2015-03-24

**Authors:** Peter Zaumseil

**Affiliations:** a IHP, Im Technologiepark 25, Frankfurt (Oder), 15236, Germany

**Keywords:** X-ray diffraction, silicon, forbidden reflections, multiple diffraction, *Umweganregung*

## Abstract

The occurrence of the basis-forbidden Si 200 and Si 222 reflections in specular X-ray diffraction ω–2Θ scans is investigated in detail as a function of the in-plane sample orientation Φ. This is done for two different diffractometer types with low and high angular divergence perpendicular to the diffraction plane. It is shown that the reflections appear for well defined conditions as a result of multiple diffraction, and not only do the obtained peaks vary in intensity but additional features like shoulders or even subpeaks may occur within a 2Θ range of about ±2.5°. This has important consequences for the detection and verification of layer peaks in the corresponding angular range.

## Abstract 2

The basis-forbidden Si 200 and Si 222 reflections are investigated in detail as a function of the in-plane sample orientation Φ and the divergence perpendicular to the diffraction plane of the used diffractometer. The results have important consequences for the detection of layer peaks near these reflections.

## 1. Introduction   

X-ray diffraction (XRD) is a well established technique to analyse the crystallographic structure of bulk materials and layers. (100)-oriented Si is the most frequently used substrate in microelectronics. To a lesser extent, (111)-oriented Si also finds application. One standard technique to detect and to study crystalline layers on such substrates is specular X-ray diffraction by ω–2Θ scans using commercially available diffractometers, usually equipped with a Cu anode. Typically, the sample alignment is done using the Si 400 or 111 reflection in such a way that during the measurement the diffraction vector is always parallel to the Si [100] or [111] direction, respectively.

Such measurements very often show a peak of the Si 200 reflection at about 2Θ = 33° for Si(100) substrates and always the Si 222 reflection at about 2Θ = 58.9° for Si(111) substrates, although both reflections are known as basis-forbidden reflections. Even though the reason for the occurrence of both reflections has been known for many decades, especially concerning the Si 200 reflection daring explanations can still be found in recent publications: for example that it occurs only when the Si lattice is distorted (Zhao *et al.*, 2005 ▶) or that it stems from the Si 400 Bragg reflection of half the wavelength of the Cu *K*α radiation (Hesse *et al.*, 2005 ▶). The origin of the appearance of forbidden reflections was discovered and theoretically described by Renninger in 1937 (Renninger, 1937 ▶). For a well defined in-plane orientation of the sample (typical Φ rotation on modern diffractometers), multiple diffraction may occur, which makes a forbidden reflection visible or increases or decreases the intensity of allowed reflections depending on Φ. For the theoretical description of this phenomenon, also known under the German word *Umweganregung*, see for example Rossmanith (2000 ▶) and references therein.

In particular, synchrotron radiation in combination with the forbidden Si or Ge 200 reflection is nowadays used to analyse problems such as the core structure of defects in Si (Richard *et al.*, 2007 ▶), the defect type in patterned Ge/Si islands (Richard, Schülli & Renaud, 2011 ▶; Richard, Malachias *et al.* 2011 ▶) and nanostructures (Kozlowski *et al.*, 2012 ▶), and the atomic ordering in Ge/Si islands (Malachias *et al.*, 2010 ▶), to mention only a few examples.

**Figure 1:**
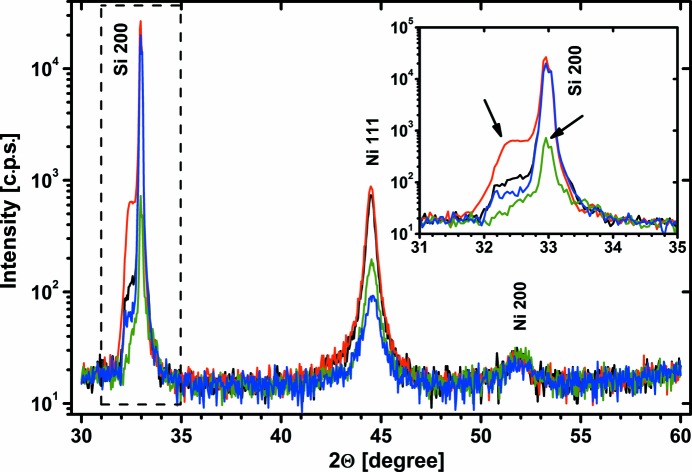
Example of ω–2Θ scans measured on four Si(100) samples with Ni deposition. The samples were placed on the stage with slightly different (±2.5°) in-plane orientation. The angular range near the Si 200 reflection is magnified in the insert. Broad side peaks and varying intensity of the Si 200 peak are marked by arrows.

Nevertheless, it seems to be worth discussing the behaviour of the Si 200 reflection for a conventional diffractometer in more detail. Since the in-plane orientation of a sample for specular ω–2Θ measurements is usually not of interest, nobody cares in this sense about obtaining an exact and repeatable sample orientation. As a result of this, the appearance of the 200 peak differs significantly from measurement to measurement. As well as the fact that the intensity of the rather sharp peak may vary over orders of magnitude down to the background level, sometimes there are broad shoulders or even subpeaks visible on both sides. Fig. 1 ▶ demonstrates this on one example. In this case, four Si(100) wafers with Ni layers deposited under different conditions were investigated with a standard X-ray diffractometer. The samples were placed on the stage considering the wafer notch such that they were roughly (±2.5°) oriented in a [110] direction. For the subject of this paper, only the range near the Si 200 peak is of interest. Broad side peaks and varying intensity of the Si 200 peak are observed and marked by arrows. While the varying intensity can be explained by the Φ dependence of multiple-diffraction phenomena, the question arises as to the reason for the broad shoulder or side peaks. Ni itself has no diffraction peak in this angular range and thus cannot be responsible for the modified Si 200 reflection, and another material should not be on the Si surface.

The most reasonable explanation is that these additional structures are also results of multiple diffraction in the Si substrate. If this is true, then the question arises, how do we distinguish between substrate-related and real layer peaks? In the case of CoSi_2_ on Si(100) (Londergan *et al.*, 2001 ▶) the detection of a clear CoSi_2_ 400 reflection confirms the existence of a (100)-oriented CoSi_2_ layer, while the identification of a peak at about 2Θ = 33° for Si 200 and CoSi_2_ 200 alone would not be sufficient. More questionable is the situation in a Gd_2_O_3_ growth study by Chaudhuri *et al.* (2014 ▶), where a broad peak at about 2Θ = 32.7° is marked as Gd_2_O_3_ 400 without any confirmation by a higher diffraction order.

In this paper, the intensity distribution in a limited angular range around the Si 200 and Si 222 reflections will be studied in detail as a function of the in-plane orientation Φ. It will be shown that the divergence of the used diffractometer perpendicular to the diffraction plane and the wavelength distribution in the incident beam have a major influence. As a conclusion of this study, a recommendation will be given for how to prove the existence of a layer peak in the vicinity of the Si 200 reflection.

## 2. Experimental details   

Standard industrial Si wafers of 100 mm diameter with (100) and (111) orientation, respectively, were used to demonstrate the behaviour of the Si 200 and Si 222 diffraction on ‘perfect’ samples.

XRD measurements were performed in parallel beam configuration on two different SmartLab diffractometers from Rigaku. The first, representing the low-resolution case, was a conventional SmartLab with a 9 kW rotating Cu anode, line focus, mirror (acting in the diffraction plane only), and 5° Soller slits on the source and detector side that define the beam divergence perpendicular to the diffraction plane. Besides the Bragg–Brentano geometry, this diffractometer configuration is typical for specular XRD measurements to analyse thin layers on Si substrates by ω–2Θ scans. The second configuration, representing the high-resolution case, was a SmartLab µHR with a 0.8 kW rotating Cu anode, micro focus, confocal Max-Flux optics and no additional Soller slits. The two mirrors of the confocal Max-Flux optics generate a beam divergence of about 0.04° not only in the diffraction plane but also perpendicular to it. With this diffractometer, supplemental measurements were performed with a Ge(400) × 2 crystal collimator added in the beam path to reduce the wavelength distribution of the incident beam nearly to the Cu *K*α_1_ line only.

Samples were aligned on an *Rx*–*Ry* stage by using the 400 or 111 reflection, respectively, so that either the normal of the (100) netplane or the normal of the (111) netplane is exactly parallel to the Φ rotation axis of the diffractometer. A conventional scintillation counter is used for all measurements.

## 3. Results and discussion   

### 3.1. Si 200 reflection   

**Figure 2:**
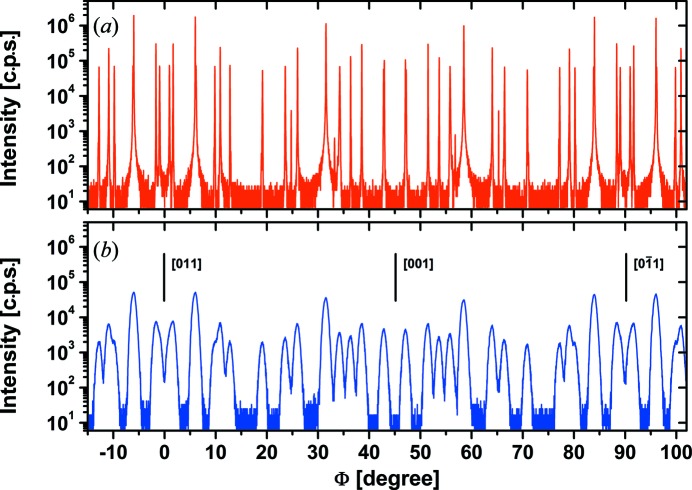
High-resolution (*a*) and low-resolution (*b*) Φ scans measured at 2Θ = 32.98°, the position of the Si 200 reflection.

The easiest way to investigate the Si 200 reflection is to select after a sample alignment on the 400 reflection its ω–2Θ position at 2Θ = 32.98° and to perform Φ scans. Such measurements are shown in Fig. 2 ▶ for both diffractometer configurations. The curve obtained in high-resolution mode (Fig. 2 ▶ *a*) indicates the occurrence of very sharp peaks reaching intensities of 10^5^–10^6^ counts per second (c.p.s.), starting from a background level of about 10 c.p.s. For comparison, the intensity reached for the 400 reflection in the same configuration is of the order of 10^7^ c.p.s. The observed background level was confirmed by ω–2Θ scans between 28 and 38° to be the general background arising from the sample under the given experimental conditions and is not especially related to the angular position of the Si 200 reflection. This indicates that Si 200 is really a forbidden reflection that shows no detectable intensity except for certain Φ orientations, where multiple diffraction (*Umweganregung*) occurs. The observed peaks have a full width at half-maximum of the order of 0.03–0.04°, which corresponds to the beam divergence perpendicular to the diffraction plane of the used diffractometer. The pattern of peaks shows a perfect mirror symmetry to the [011] and [001] directions, as expected for the lattice structure of Si.

The measurement in low-resolution mode (Fig. 2 ▶ *b*) shows a similar peak pattern but strongly smeared out so that details get completely lost and the intensity between some adjacent peaks does not reach the background level. This explains why the intensity of the Si 200 peak varies from sample to sample as long as the in-plane orientation of the sample is not exactly reproduced. Under the given low-resolution mode representing typical diffractometer conditions, the intensity of the Si 200 reflection may vary between the background level and more than 10^4^ c.p.s. If the sample is approximately aligned in the [011] direction as in the case demonstrated in Fig. 1 ▶, the Si 200 reflection is always detected within a Φ range of ±3°, and a variation in Φ by 1° is sufficient to modify the peak intensity by more than one order of magnitude.

The Φ scans in Fig. 2 ▶ deliver furthermore a clear argument against the explanation of the Si 200 peak as a higher harmonic, *e.g.* Si 400, reflection with half the Cu *K*α wavelength from the continuous spectrum. If this were true then a constant intensity should be visible, independent of the in-plane sample orientation. Furthermore, as long as no X-ray optical element is used in the beam pass in front of the sample (as typical for the Bragg–Brentano setting), the Si 400 reflection would contribute to the background level at any Bragg angle around the Si 200 peak position owing to the corresponding wavelength from the continuous spectrum. The background level and the contribution of the Si 400 reflection in the given Φ scans in Fig. 2 ▶, as well as in ω–2Θ scans near the 200 peak, are clearly below 10 counts per second, which is orders of magnitude lower than the observed peaks.

**Figure 3:**
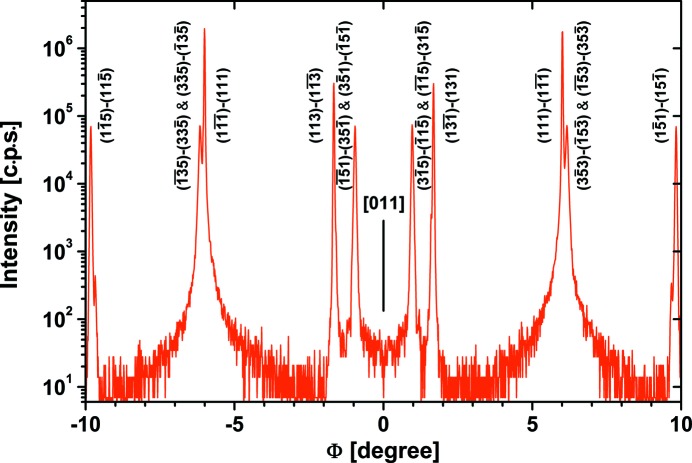
High-resolution Φ scan close to the [011] in-plane direction with indication of the associated first and second reflecting planes of Si 200 multiple diffraction (*Umweganregung*).

Fig. 3 ▶ shows a fraction of the high-resolution curve of Fig. 2 ▶(*a*) around the [011] direction with an indication of the associated first and second reflecting planes of the Si 200 multiple diffraction as calculated by Hwang (2001 ▶). The calculated and measured peak positions agree perfectly, with errors less than the measuring step width of 0.012°.

**Figure 4:**
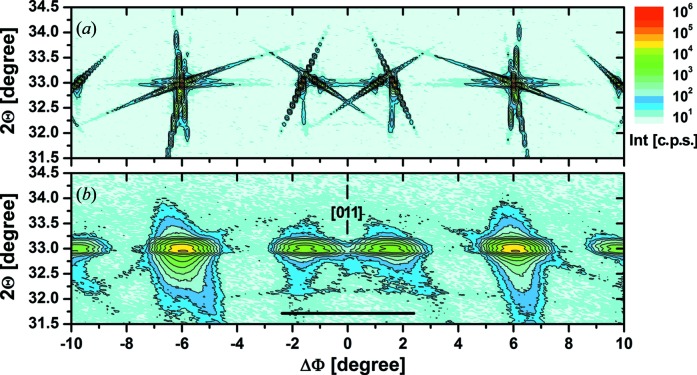
2Θ–Φ mapping of the Si 200 reflection near the [011] in-plane direction measured (*a*) in high-resolution and (*b*) in low-resolution mode. The dotted structure of some streaks is caused by the finite step widths of 0.02 and 0.1° for 2Θ and Φ, respectively. The bar between ΔΦ = ±2.5° marks approximately the Φ range within which the measurements shown in Fig. 1 ▶ were performed.

**Figure 5:**
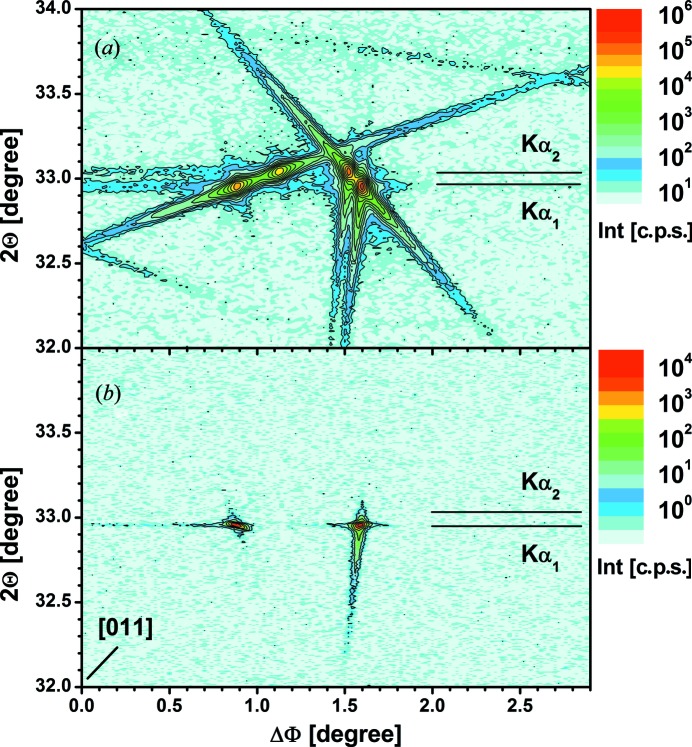
2Θ–Φ mapping of the Si 200 reflection near the [011] in-plane direction measured in high-resolution mode and (*a*) parallel beam configuration and (*b*) with an additional Ge(400) × 2 collimator crystal and step widths of 0.02 and 0.02° for 2Θ and Φ, respectively.

The more complex diffraction behaviour becomes visible in a 2Θ–Φ mapping, as shown in Fig. 4 ▶(*a*) for the high-resolution case and the same Φ range as in Fig. 3 ▶. Not only are there intensity peaks visible at the exact Bragg position of the Si 200 reflection, but inclined intensity streaks reach 2Θ values ±1.5° away from the peak position. It is necessary to note that the dotted structure of some streaks is caused by finite step widths of 0.02 and 0.1° for 2Θ and Φ, respectively. In agreement with calculations by Rossmanith *et al.* (2001 ▶), these streaks are caused by the participation of a certain wavelength interval in the diffraction, since in the used parallel beam configuration there was no crystal collimator involved to suppress the Cu *K*α_2_ line and other adjacent wavelengths from the continuous spectrum. This becomes clear in Fig. 5 ▶(*a*), where a 2Θ–Φ mapping of a smaller area near the [011] in-plane direction is repeated with lower step width in Φ. The expected angles of the Si 200 reflection for Cu *K*α_1_ and *K*α_2_ radiation are marked. Corresponding intensity maxima can clearly be identified, lying exactly on the observed streaks. Fig. 5 ▶(*b*) shows the same mapping for conditions where the incident wavelength distribution is limited by the use of a crystal collimator to the Cu *K*α_1_ line only. As expected, the observed intensities are much lower, but intensity peaks can now be found at the angular position for *K*α_1_ only, and all wavelength-related streaks have disappeared. The weak, nearly perpendicular streak visible at the peak near ΔΦ = 1.6° that is also visible for the parallel beam case is most likely related to the slit-limited angular acceptance of the detector.

Coming back to the more intense parallel beam configuration without collimator crystal, the consequence for ω–2Θ scans on Si with (100) orientation under these high-resolution conditions is that depending on the Φ position of the sample not only one sharp peak at the exact Bragg position of the Si 200 reflection may occur, but two or even three sharp peaks distributed over a wider angular range are possible.

The 2Θ–Φ mapping under low-resolution conditions shown in Fig. 4 ▶(*b*) indicates a very diffuse intensity distribution. Nevertheless, the now smeared out streaks of multiple diffraction cause a characteristic intensity pattern, and it becomes clear that the Si 200 peak not only may appear or disappear but also may have shoulders or even broad subpeaks on both sides depending on the in-plane orientation of the sample. The bar between ΔΦ = ±2.5° marks approximately the Φ range within which the measurements shown in Fig. 1 ▶ were performed. The broad intensity shoulder on the low-angle side of the 200 reflection marked in Fig. 1 ▶ is clearly caused by the Si substrate itself, and it is not the reflection of any kind of surface layer. Likewise, the variation of the height of the 200 reflection can be easily explained by a slight modification of the sample orientation.

### 3.2. Si 222 reflection   

**Figure 6:**
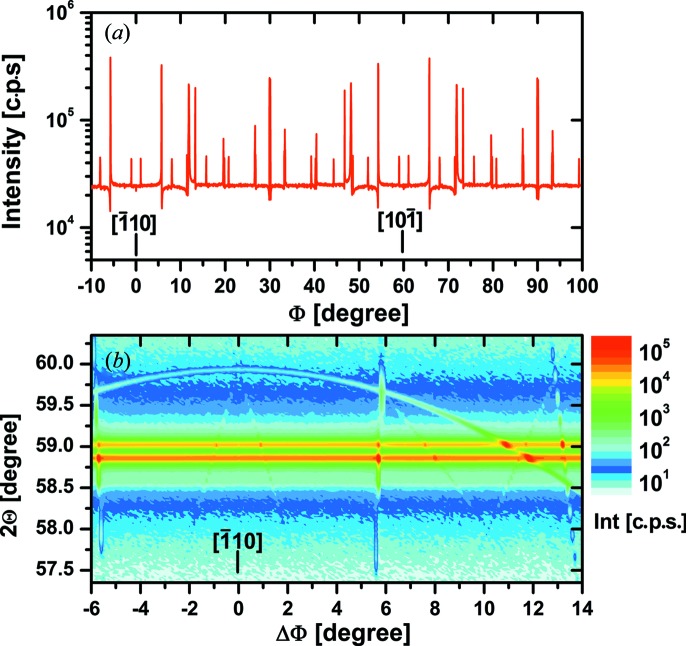
Φ scan measured at 2Θ = 58.86°, the position of the Si 222 reflection (*a*), and 2Θ–Φ mapping of the Si 222 reflection near the 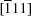 in-plane direction (*b*) measured in high-resolution mode.

The situation for the Si 222 reflection is rather different from Si 200. Fig. 6 ▶(*a*) shows a Φ scan measured at the Bragg angle of the Si 222 reflection (2Θ = 58.86°) in high-resolution mode. Here a constant intensity of about 2.5 × 10^4^ c.p.s. exists independent of Φ, which is interrupted by sharp peaks generated by multiple diffraction (*Umweganregung*) that are in most cases associated with a dip to lower intensity.

The 2Θ–Φ mapping near the 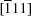 in-plane direction shown in Fig. 6 ▶(*b*) confirms the constant intensity band of the Si 222 reflection clearly split into *K*α_1_ and *K*α_2_ lines. Some streaks and even a bow caused by multiple diffraction are visible, but they seem to be less pronounced because of the generally higher level of background intensity.

The fact that most basis-forbidden reflections do not have precisely zero intensity can be explained (Tischler *et al.*, 1988 ▶) by noncentrosymmetric parts in the atomic charge distribution, which produces a nonzero structure factor for the 222, 442, 622 and other basis-forbidden reflections. But, for the 200 reflection, where the atomic charge distribution is consistent with the atomic site geometry, the structure factor is exactly zero.

## 4. Summary and conclusions   

It was demonstrated that the occurrence of the basis-forbidden Si 200 reflection in ω–2Θ scans is caused by multiple diffraction (*Umweganregung*), and the intensity and peak shape depend on three parameters: the in-plane orientation of the sample Φ, the divergence of the used diffractometer perpendicular to the diffraction plane and the wavelength distribution in the incident beam. The intensity can vary between really zero and values close to non-forbidden reflections. For diffractometers with low divergence perpendicular to the diffraction plane the 200 reflection may be represented by no or up to three relatively sharp peaks that show up in a 2Θ range between about 31 and 35°. For more commonly used diffractometers with a divergence perpendicular to the diffraction plane of the order of some degrees, no matter whether they are used in parallel beam or Bragg–Brentano geometry, the 200 reflection may exhibit shoulders or subpeaks in the same angular range as mentioned above, or in extreme cases a broad peak might even occur somewhere in this angular range, only without any sharp peak at the exact 2Θ value.

This behaviour of the Si substrate itself has significant consequences for the analysis of layer materials on Si(100) substrates that show diffraction peaks in the 2Θ range between 31 and 35°, and the following recommendation can be given. For diffractometers with low divergence perpendicular to the diffraction plane the problem is minor, since a broad diffraction peak from a thin layer can easily be distinguished from the rather sharp Si 200 peak (or multiple peaks). The situation becomes more critical for standard X-ray diffractometers with relatively large divergence perpendicular to the diffraction plane. Although the divergence can be reduced by the use of Soller slits with low angular acceptance, this is usually not done since it is always associated with a significant intensity reduction. To avoid under these conditions any confusion between substrate-related diffraction phenomena and real layer diffraction it is urgently necessary to check the behaviour of an observed peak by rotation in Φ. Only if the peak intensity does not change (or at least does not vanish) depending on Φ can one be sure that this peak is really related to a layer material. A second alternative is the use of a crystal collimator, but this is in many cases not acceptable owing to the strong reduction of intensity associated with that approach.

Taking the results of these investigations into account, the proof of epitaxial Gd_2_O_3_ growth with (100) orientation on Si(100) substrates as reported by Chaudhuri *et al.* (2014 ▶), based on ω–2Θ scans on three samples without any detailed information about the exact experimental conditions, cannot be accepted. The observed shoulder on the low-angle side of the Si 200 reflection peak, which was indexed as the Gd_2_O_3_ 400 reflection, is very similar to that shown in Fig. 1 ▶. Only a Φ scan at this 2Θ position is able to give final evidence for the growth of (100)-oriented Gd_2_O_3_.
